# Diagnosis of Imported Monkeypox, Israel, 2018

**DOI:** 10.3201/eid2505.190076

**Published:** 2019-05

**Authors:** Noam Erez, Hagit Achdout, Elad Milrot, Yuval Schwartz, Yonit Wiener-Well, Nir Paran, Boaz Politi, Hadas Tamir, Tomer Israely, Shay Weiss, Adi Beth-Din, Ohad Shifman, Ofir Israeli, Shmuel Yitzhaki, Shmuel C. Shapira, Sharon Melamed, Eli Schwartz

**Affiliations:** Israel Institute for Biological Research, Ness-Ziona, Israel (N. Erez, H. Achdout, E. Milrot, N. Paran, B. Politi, H. Tamir, T. Israely, S. Weiss, A. Beth-Din, O. Shifman, O. Israeli, S. Yitzhaki, S.C. Shapira, S. Melamed);; Shaare-Zedek Medical Center, Jerusalem, Israel (Y. Schwartz, Y. Wiener-Well);; Tel Aviv University, Tel Aviv, Israel (E. Schwartz)

**Keywords:** Monkeypox, outbreak, monkeypox virus, orthopoxvirus, transmission electron microscopy, zoonoses, disease outbreaks, viruses, Israel, West Africa

## Abstract

We report a case of monkeypox in a man who returned from Nigeria to Israel in 2018. Virus was detected in pustule swabs by transmission electron microscopy and PCR and confirmed by immunofluorescence assay, tissue culture, and ELISA. The West Africa monkeypox outbreak calls for increased awareness by public health authorities worldwide.

Monkeypox is a zoonotic disease caused by monkeypox virus, an orthopoxvirus closely related to variola virus, the causative agent of smallpox. Human cases were first described in 1970; in subsequent decades, sporadic outbreaks were reported in Africa. Mortality rates are 1%–10% (*1*,*2*). The 2 clades, Congo-Basin and West African, each cause disease; the West African clade is considered to be less virulent and is associated with a lower mortality rate (*3*). Nevertheless, this clade is responsible for the largest documented monkeypox outbreak in West Africa (132 confirmed cases in Nigeria) (*4*). Human infection with monkeypox occurred in the United States in 2003, when imported animals from Africa infected pet prairie dogs (*5*). In September 2018 in the United Kingdom, 2 imported cases of monkeypox, were detected in persons from Nigeria (*6*); one of these cases caused nosocomial infection of a healthcare worker (HCW). We report a case of monkeypox in Israel.

## The Study

On October 4, 2018, a 38-year-old man sought care for generalized rash and fever at the Department of Emergency Medicine at Shaare-Zedek Medical Center, Jerusalem, Israel. This Israel resident had returned from Port Harcourt, Rivers State, Nigeria, where he had worked a desk job for the previous 10 years. On September 17, during his last trip to Nigeria, he had disposed of 2 rodent carcasses at his residence. He returned to Israel on September 23 and on September 29 noticed 2 itchy lesions on his penis shaft. The next day, he had fever (38.8°C) and chills and started self-medicating with nonsteroidal antiinflammatories and oral penicillin. On October 1, an erythematous rash appeared first on his face and later on his trunk and extremities. 

**Figure 1:**
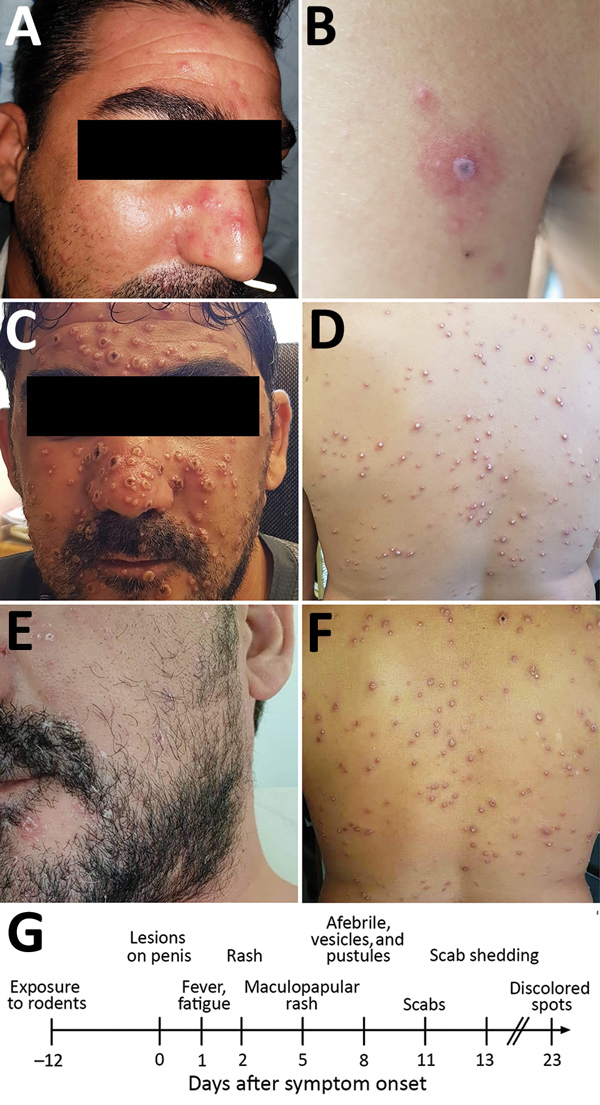
Dermal manifestations of monkeypox on patient in Israel, 2018. Maculopapular rash was apparent on the face (A) and body on the day of hospital admission. A lesion on the left proximal extremity (B) was suspected to be a rickettsial eschar. After 3 days, the rash changed into vesicles and pustules on the face (C) and body (D). Skin resolution was apparent 13 days after admission; pustules and vesicles crusted and were shed (E, F). G) Timeline of disease progression.

Examination at Shaare-Zedek Medical Center on October 4 revealed that the patient was febrile and had a nonblanching maculopapular rash on his face (Figure 1, panel A), neck, trunk, and lower and upper extremities; several lesions on his palms and soles; 2 ulcers with an erythematous base on his penis shaft; and bilateral enlarged and tender lymph nodes in his groin. Blood test results indicated moderate thrombocytopenia (98,000 platelets/μL) and mild hepatitis. One lesion on the posterior aspect of his left arm (Figure 1, panel B) was suspected to be an eschar, raising the possibility of rickettsialpox. The patient was therefore hospitalized and administered oral doxycycline. His condition improved, and the next day he was discharged with doxycycline and instructions to remain isolated at home.

At a follow-up visit 2 days later (October 7), he was afebrile. The rash was locally synchronous and had progressed from maculopapular to vesicular and pustular; some lesions displayed black umbilication and crusting (Figure 1, panels C, D). Oral examination revealed bilateral tonsillar enlargement and ulcers in the posterior pharynx. Serology results were positive for varicella IgG (past infection) and negative for Coxiella burnetii, *Rickettsia conori*, *Rickettsia typhi*, *Brucella *spp., Treponema pallidum*,* and antigen/antibody combination for HIV. Pustular samples were negative for herpes simplex virus by PCR. Because of the rash characteristics and the patient’s travel history, monkeypox was suspected.

Samples were sent to the Israel Institute for Biological Research, Ness-Ziona, Israel, and processed in Biosafety Level 3 laboratories. The pustule sample was processed for PCR analysis and transmission electron microscopy. Vero cells were infected for immunofluorescence assay and monitored for cytopathic effect. For transmission electron microscopy, particles were enriched by using a Beckman Airfuge (https://www.beckman.com) before negative staining with phosphotungstic acid. 

**Figure 2:**
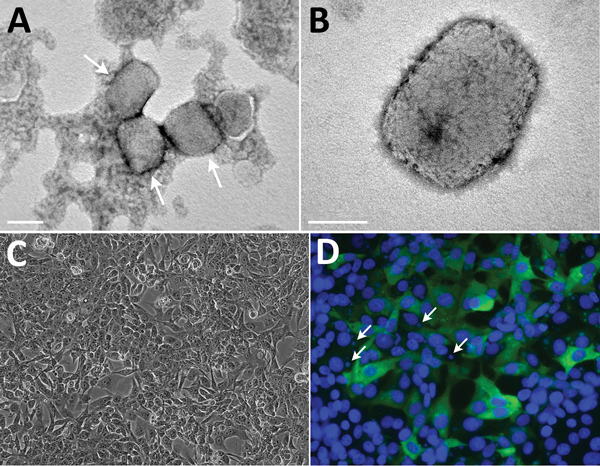
Transmission electron microscopy and cell culture–based diagnosis of monkeypox in patient in Israel, 2018. Virus particles were detected in lesion samples as either virion aggregates (arrows) (A) or individual virions (B) with a typical brick shape. Infected Vero cells depicted typical cytopathic effect, exhibiting cell detachment and rounding. Scale bar in A indicates 0.2 μm; scale bar in B indicates 100 nm. C) Infected Vero cells depicting typical cytopathic effect: cell detachment and rounding. Original magnification ×10. D) Immunofluorescent staining of infected Vero cells: DNA (DAPI [4′,6-diamidino-2-phenylindole] stain) and monkeypox virus; viral factories are evident (arrows). Original magnification ×25.

The sample exhibited numerous brick-shaped particles, characteristic of orthopoxviruses. Particles were observed to be in clusters (up to 10 virions in each cluster) embedded in skin tissue and as single virions (Figure 2, panels A, B). Viral particle dimensions (± SD) were 281 ± 18 nm × 220 ± 17 nm (n = 24), in accordance with previously reported dimensions for monkeypox virus (*5*).

PCR diagnosis was based on specific primers to discriminate between the West African (581 bp) and the Congo-Basin (832 bp) clades by product size (*7*). The PCR product size corresponded to that of the West African clade currently circulating in Nigeria (*8*). This finding was confirmed by high-throughput sequencing.

Within 24 hours of infection, cytopathic effect was observed in Vero cells, exhibiting typical monolayer separation and cell rounding (Figure 2, panel C). The result of immunofluorescence assay with a specific antibody against orthopoxviruses was positive; some cells exhibited viral factories, typical for orthopoxvirus infection (Figure 2, panel D) (*9*).

The patient was instructed to remain isolated in his residence until he had fully recovered. Days after he returned home, the pustules turned to scabs (0.3–0.8 mm in diameter) and were shed (Figure 1, panels E, F). Concomitant with recovery, antibodies against orthopoxvirus and a neutralizing antibody titer (50% plaque reduction neutralization test titer = 134) developed, comparable to those of smallpox-vaccinated humans (*10*). Of note, scabs collected from the patient during recovery, then homogenized and tested for monkeypox virus, contained viable viral loads of 10^5^–10^7^ PFU/scab.

All of the patient’s contacts in Israel (5 household members and 11 HCWs) were offered smallpox vaccination, but only 1 HCW agreed. All contacts were followed up for 21 days; no virus transmission was detected.

## Conclusions

Since the first documented case of human monkeypox in 1970, sporadic outbreaks have been reported, especially in the Congo Basin and West Africa. Contributing to the increased frequency of such occurrences were discontinued vaccination against smallpox, increased interaction with wildlife because of deforestation and population movement, consumption of bushmeat, and increased population density (*11*,*12*). Although most infections are acquired from wildlife, human-to-human transmission has been reported, as in the 1996–1997 outbreak in the Democratic Republic of the Congo (*13*) and the current outbreak in West Africa (*8*). The availability and speed of international transportation combined with the natural progression of the disease (long incubation and prodromal periods, up to 21 days combined) increase the risk for monkeypox spread from rural regions into urban areas and to countries outside Africa. Indeed, during September and October 2018, monkeypox was diagnosed in the United Kingdom and Israel (*6*,*14*).

Thus far, all imported cases of monkeypox in humans (United States in 2003, United Kingdom and Israel in 2018) have involved the West African clade of the virus (*3*,*6*). After a similar incubation period (12 days), all patients had fever and chills, lymphadenopathy, and skin lesions (*5*,*6*). Although the patient in Israel had numerous vesiculopustules on his face and body, the patients involved in the US outbreak had substantially fewer (1–50) and reported a persistent cough, which the patient from Israel did not report. Of note, the first sign noted by the Israel and UK patients was groin lesions (*6*). Although past reports considered the Congo Basin clade to be more virulent (*2*,*3*,*12*), recent reports show that the West African clade can also cause disseminated disease and can be transmitted from human to human (*4*,*8*).

For this study, we used multiple diagnostic approaches. The virus was detected in pustule swab specimens by transmission electron microscopy and PCR within 3 hours of sample arrival and confirmed by immunofluorescence assay, tissue culture, and ELISA for orthopoxvirus antigens.

The very high virus titers contained by pustules and scabs, as demonstrated in this case, increase the risk for human-to-human transmission and environmental spread. To prevent further transmission, HCWs should implement safety practices and local authorities should map contacts and consider use of smallpox vaccines or antiviral drugs (*14*,*15*), according to risk assessment.
